# Editorial: Non-canonical NF-κB signaling in immune-mediated inflammatory diseases and malignancies

**DOI:** 10.3389/fimmu.2023.1252939

**Published:** 2023-07-26

**Authors:** Sander W. Tas, Vanessa L. Bryant, Matthew C. Cook

**Affiliations:** ^1^Department of Rheumatology and Clinical Immunology, Academic Medical Center, Amsterdam, Netherlands; ^2^Department of Experimental Immunology, Amsterdam University Medical Center, Amsterdam, Netherlands; ^3^Immunology Division, Walter and Eliza Hall Institute of Medical Research (WEHI), Parkville, VIC, Australia; ^4^Department of Medical Biology, The University of Melbourne, Parkville, VIC, Australia; ^5^Department of Clinical Immunology and Allergy, The Royal Melbourne Hospital, Parkville, VIC, Australia; ^6^Cambridge Institute for Therapeutic Immunology and Infectious Diseases, University of Cambridge, Cambridge, United Kingdom; ^7^John Curtin School of Medical Research, Australian National University, Canberra, ACT, Australia

**Keywords:** nuclear factor-κB, non-canonical, IMID (immune-mediated inflammatory diseases), malignancies, signaling/signaling pathways

In thirty years since its discovery as a regulator of transcription in B cells, the NF-κB family of transcription factors has been demonstrated to mediate activation and differentiation of many components of immunity, and has also been shown to have actions in other cell types and diverse tissues (1). Genetic approaches to discover and characterize the components of the NF-κB family, including the receptors and proximal signaling events that liberate NF-κB for nuclear translocation, as well as the transcriptional events downstream of NF-κB activation, have been elucidated through cloning, deletion studies, and most recently through identification and characterization of inborn errors of immunity in humans. The last approach has provided new insights into human disease since hypomorphic and gain-of-function missense mutations confer phenotypes distinct from those conferred by gene deletion in model systems (2–5). NF-κB proteins are important regulators of immunity via their collective coordination of the transcription or repression of >500 genes (encoding cytokines, chemokines, apoptosis factors), involved in proliferation, inflammation, cell development, survival, and immune cell function. Activation of NF-κB can occur via two main signaling cascades: the NF-κB1, or canonical pathway, and the NF-κB2, or non-canonical pathway (**Figure 1**). Compared with the canonical pathway, which regulates downstream signaling in multiple immune cells, expression of components of the non-canonical NF-κB pathway is more restricted, and mostly limited to triggering of specific receptors, including CD40, BAFF-R, and lymphotoxin-β receptor (LTβR). Activation of NF-κB depends on processing of IκB molecules, which liberates NF-κB family members for nuclear translocation. Both NF-κB1 (p105) and NF-κB2 (p100) behave as IκB molecules, and after phosphorylation, they undergo cleavage, resulting in the formation of their active subunits (p50 and p52, respectively). These subunits form transcriptionally active heterodimers with other NF-κB members (RelA/p65, RelB, and c-Rel), and inhibitory homodimers. Inborn errors of canonical and non-canonical NF-κB signaling have been reported and mutations in NF-κB2 signaling components do not often cause lethality, but result in immune pathology, including B-cell lymphopenia or lymphoma, autoimmunity, and immune deficiency (3, 6–9).

**Figure 1:**
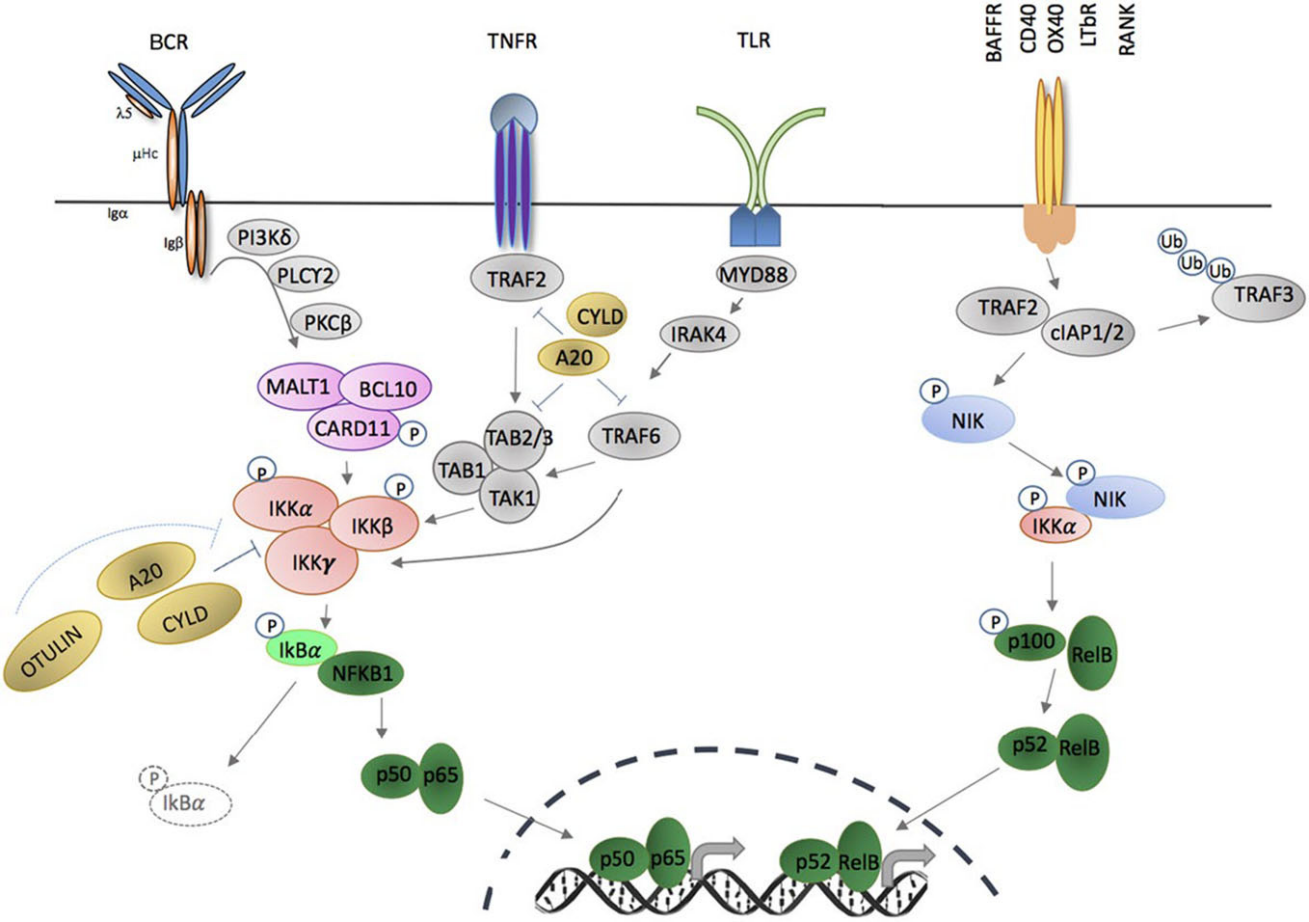
Activation of canonical and non-canonical NF-KB signaling pathways through membrane- bound extracellular ligands. TNFR and toll-like receptor (TLR) family members, as well as antigen receptors activate the canonical pathway; and regulation of B cell activating factor receptor (BAFFR), CD40, OX40, LTBR, and receptor activator of nuclear factor KB (RANK) activate the non-canonical pathway. Triggering of canonical pathway results in phosphorylation of full length NF-KB1 (p105) and cleavage to its active subunit, p50 and formation of p50/p65 (RelA) heterodimers. Activation via the non-canonical signaling pathway leads to cleavage of p100 to p52 active subunit and formation of p52/RelB complexes. Both pathways require phosphorylation and activation of inhibitor of KB kinase (IKK) subunit(s) in order to release NF-kB molecules that are constitutively sequestered by an inhibitor, e.g., IκBa. Phosphorylation and ubiquitination of the inhibitors by IKKS release NF-kB allowing nuclear translocation of homodimers or heterodimers complexes and binding to the KB site of their target genes (adapted from: Miraghazadeh & Cook, Front. Immunol 2018).

The aim of this Research Topic was to gather contributions providing the context of recent progress in understanding non-canonical NF-κB contributions to pathology by summarizing activation, regulation, signaling, and outcomes of the non-canonical pathway. In addition, we provided space for recent advances on the importance of non-canonical NF-κB signaling in pathological processes involved in immune-mediated inflammatory diseases, including the actions of this pathway in both stromal cells and immune cells. Finally, we created an opportunity to discuss how these advances might result in new treatment approaches.

In this Research Topic we have collected several articles that provide insight into the importance of non-canonical NF-κB signaling both in healthy conditions and, particularly, in disease settings.

Fan et al. investigated the importance of TRIM proteins in the regulation of NF-κB signaling and identified TRIM67 as a negative regulator of TNF-induced NF-κB signaling. Overexpression of TRIM67 resulted in reduced IκBα degradation, which was accompanied by reduced nuclear translocation of p65. This resulted in a significant decrease in pro-inflammatory cytokine production (i.e. IL-6 and TNF), whereas genetic deletion of TRIM67 in primary mouse embryonic fibroblasts resulted in enhanced pro-inflammatory cytokine production. The underlying mechanism of TRIM67-mediated negative regulation of NF-κB signaling involved competitive binding of TRIM67 to β-TrCP and thereby caused inhibition of β-TrCP mediated IκBα degradation. Further studies are required to determine the relevance of this mechanism to the pathophysiology of chronic inflammatory diseases and/or infections.

In an unconventional study, Tang et al. identified an important pro-inflammatory role for cell-free hemoglobin in hemolytic disease of grass carp (*C. idella*). Using three forms of hemoglobin (Hb, MetHb, and FerrylHb) the group demonstrated that in a sterile hemolysis model, each form increased production of reactive oxidative species, causing oxidative damage and apoptosis, as well as the expression of inflammation-related genes via NF-κB signaling. Interestingly, this process could be inhibited by caffeic acid phenethyl ester, which has previously been demonstrated to block canonical NF-κB signaling. Whether these mechanisms are also at play in hemolysis in the human setting remains to be tested.

In a translational study, Jeucken et al. demonstrate differential roles of canonical and non-canonical NF-κB signaling in CD4^+^ memory T cells (Tm) induced activation of endothelial cells (ECs). They performed RNA sequencing of ECs stimulated with supernatants of Tm and observed activation of both pathways. However, treatment of ECs with highly specific IKKβ or NIK inhibitors resulted in differential effects. The functional consequences of the inhibitors were subsequently tested in trans-endothelial migration assays with neutrophils in relation to currently employed therapies for immune-medicated inflammatory diseases (IMIDs) such as rheumatoid arthritis and vasculitis. Both IKKβ and NIK inhibition resulted in a strong inhibitory effect which could potentiate the effects of several biologics and JAK/STAT-inhibitors. These findings provide a rationale for (combination) treatment with these small molecule inhibitors as a novel treatment strategy for immune-mediated inflammatory diseases (IMIDs) in which there remains unmet medical need.

The current state of knowledge regarding the involvement of NIK aberrations in B cell malignancies and the therapeutic potential of targeting NIK is summarized by Haselager and Eldering. First, they discuss the regulation of NIK and the extrinsic and intrinsic factors that contribute to elevated NF-κB activity in B cell malignancies. Next, they highlight the role of NIK in crosstalk between the canonical and the non-canonical pathways. Lastly, they give an overview of small molecule NIK inhibitors tested in vitro and in vivo, and speculate on the benefits and potential risks of NIK inhibition in hematology patients.

Finally, Aoki et al. investigated the effects of a loss-of-function mutation in the *Map3k14* gene encoding NIK in a mouse model of periodontitis. They found that inflammation-induced bone resorption was suppressed due to a decreased number of osteoclasts in these mice. Using local administration of a pharmacological NIK inhibitor, they established that osteoclast formation and bone resorption was inhibited when the model was applied to *wildtype* mice. Therefore, they conclude that NIK-mediated non-canonical NF-κB signaling may be a novel therapeutic target in periodontal disease.

In conclusion, this Research Topic comprises a diverse collection of papers on the role of (non-canonical) NF-κB signaling in distinct pathological processes, providing a strong rationale for development of NIK targeting therapies in autoimmunity, periodontal disease, and B cell malignancies. At the same time, many questions still remain unanswered and it is clear that more research is required to uncover the ins and outs of (non)canonical NF-κB signaling in health and disease.

## Author contributions

ST has drafted the manuscript. VB and MC provided important input. All authors revised the manuscript for important intellectual content and approved the submitted version.
